# C1Q Assay Results in Complement-Dependent Cytotoxicity Crossmatch Negative Renal Transplant Candidates with Donor-Specific Antibodies: High Specificity but Low Sensitivity When Predicting Flow Crossmatch

**DOI:** 10.1155/2016/2106028

**Published:** 2016-09-04

**Authors:** José M. Arreola-Guerra, Natalia Castelán, Adrián de Santiago, Adriana Arvizu, Norma Gonzalez-Tableros, Mayra López, Isaac Salcedo, Mario Vilatobá, Julio Granados, Luis E. Morales-Buenrostro, Josefina Alberú

**Affiliations:** ^1^Department of Transplantation, National Institute of Medical Sciences and Nutrition Salvador Zubirán, Vasco de Quiroga 15, Colonia Belisario Domínguez, Sección XVI, Tlalpan, 14080 Mexico City, Mexico; ^2^Histocompatibility Laboratory, National Institute of Medical Sciences and Nutrition Salvador Zubirán, Vasco de Quiroga 15, Colonia Belisario Domínguez, Sección XVI, Tlalpan, 14080 Mexico City, Mexico; ^3^Department of Nephrology-Mineral Metabolism, National Institute of Medical Sciences and Nutrition Salvador Zubirán, Vasco de Quiroga 15, Colonia Belisario Domínguez, Sección XVI, Tlalpan, 14080 Mexico City, Mexico

## Abstract

The aim of the present study was to describe the association of positive flow cross match (FXM) and C1q-SAB.* Methods*. In this observational, cross-sectional, and comparative study, patients included had negative AHG-CDC-XM and donor specific antibodies (DSA) and were tested with FXM. All pretransplant sera were tested with C1q-SAB assay.* Results*. A total of 50 donor/recipient evaluations were conducted; half of them had at least one C1q+ Ab (*n* = 26, 52%). Ten patients (20.0%) had DSA C1q+ Ab. Twenty-five (50%) FXMs were positive. Factors associated with a positive FXM were the presence of C1q+ Ab (DSA C1q+ Ab: OR 27, 2.80–259.56, *P* = 0.004, and no DSA C1q+ Ab: OR 5, 1.27–19.68, *P* = 0.021) and the DSA LABScreen-SAB MFI (OR 1.26, 95% CI 1.06–1.49, *P* = 0.007). The cutoff point of immunodominant LABScreen SAB DSA-MFI with the greatest sensitivity and specificity to predict FXM was 2,300 (sensitivity: 72% and specificity: 75%). For FXM prediction, DSA C1q+ Ab was the most specific (95.8%, 85–100) and the combination of DSA-MFI > 2,300 and C1q+ Ab was the most sensitive (92.0%, 79.3–100). *Conclusions*. C1q+ Ab and LABScreen SAB DSA-MFI were significantly associated with FXM. DSA C1q+ Ab was highly specific but with low sensitivity.

## 1. Introduction

An almost 50-year-long history has been written on the search for a clinically relevant assay that could predict antibody mediated graft injury. The story dates back to 1969, when Patel and Terasaki demonstrated for the first time the highly significant correlation between a positive complement-dependent cytotoxicity (CDC) crossmatch (XM) assay and hyperacute or accelerated acute graft rejection in kidney transplant recipients [1]. Ever since, it has been generally accepted that a positive CDC-XM is a contraindication to transplantation. The assay, however, is insensitive for the detection of low HLA donor-specific alloantibody (DSA) levels.

The newer solid phase assay, Luminex Single Antigen Beads (SAB) assay, is highly sensitive and specific for the target antigen when compared with the CDC assay but less predictive of transplant outcome since it detects both complement-binding (pathogenic antibodies) and less clinically relevant, non-complement fixing anti-HLA antibodies (HLA-Abs) [2, 3]. Additionally, immunoglobulin G (IgG) flow cytometry crossmatch (FXM) is very sensitive but cannot differentiate complement fixing antibodies (CFAb) from non-CFAb. Also, the debate persists on whether the correct mean fluorescence intensity (MFI) cutoff value has been determined to establish DSA positivity or for correlation with FXM results [4, 5]. Therefore, every Histocompatibility Laboratory determines its own threshold to identify pretransplant, clinically relevant antibodies, or posttransplant DSA that warrant therapeutic intervention [6].

The fundamental description of complement split fragment C4d capillary deposition in the biopsies of patients with early renal graft function deterioration suggesting humoral alloreactivity [7], as well as their correlation with* de novo* DSA at the time of renal allograft dysfunction [8], evidenced the injury induced by the interaction of endothelium-bound antibodies and complement activation. The C4d FlowPRA assay was subsequently developed to assist in the diagnosis of antibody mediated rejection (AMR) through the detection of circulating complement fixing antibodies (CFAbs) [9–12]. The assay correlated with C4d deposition in graft biopsies and showed a high level of specificity (0.92 (95% CI: 0.86–0.98)) exceeding that calculated by IgG FlowPRA; however, the assay has lower sensitivity compared to IgG FlowPRA screening [11]. The mean fluorescence intensity (MFI) in the C4d assay seems to be in the range of 500 to 3500 and C4d-fixing capability of low level DSA is not predictive of early AMR [13, 14]. However, the use of C4d FlowPRA for pretransplant screening of kidney transplant recipients (KTR) showed complement fixing presensitized patients to have significantly worse graft survival than those with non-complement fixing [15].

Considering the important role of complement fixation in AMR and its detrimental effect on graft outcome, the need for an assay distinguishing CFAb from non-CFAb, with high sensitivity and specificity, was paramount, particularly to determine the immediate posttransplant risk in highly sensitized patients. To fulfill this need for identifying CFAb that can bind the first component of complement (C1q), a solid phase one-step C1q-SAB assay was developed by the Histocompatibility Laboratory at Stanford University [16].

The same group developed a new combo-flow crossmatch by integrating standard IgG-FXM with C1q-FXM (CFXM-IgG), to simultaneously detect donor-specific IgG Ab and CFAb in a single reaction [17]. The results obtained in 83 samples demonstrated good correlation between CFXM-IgG and the regular IgG-FXM. Moreover, the new assay was able to identify additional positive XMs that had previously tested as negative by CDC-XM and in the presence of DSA determined by SAB [17]. The C1q technology was licensed to One Lambda, Inc. (Canoga Park, CA), and they commercialized C1q-SAB for Luminex.

This study describes the gamut of pretransplant HLA-Ab in patients who were evaluated by FXM because they had DSA by Luminex-SAB (LABScreen SAB) and negative AHG-CDC-XM. It also evaluates the association and the predictive capacity of some antibodies' characteristics (antigenic specificity, MFI, and capacity to bind C1q, among others) and the FXM result.

## 2. Patients and Methods

This is an observational, cross-sectional, comparative, and single center study. All the patients included in this study had a negative AHG-CDC-XM but DSA against their potential living donors (as detected by Luminex-SAB); then according to institutional protocol all of them were tested with FXM. For this study, the frozen sera samples were tested with the C1q-SAB.

Forty-one renal transplant candidates were evaluated with their respective potential living donors; five of them had 2 potential donors and 2 of them had 3 potential donors, so a total of 50 events were analyzed.

The Histocompatibility Laboratory at our institution is a reference center for pretransplant testing of patients from different transplant centers in Mexico, and most of the patients included in this report were referrals from other national centers.

*HLA Typing*. HLA typing is obtained for A, B, C, DR, and DQ antigens by molecular biology techniques (Micro SSP*™* HLA DNA Typing Trays, One Lambda, Canoga Park, CA).

*Crossmatch*. In our setting, XM is performed by direct CDC-XM on total and separated T and B lymphocytes. T and B cells are isolated with magnetic beads (FluoroBeads®, One Lambda, Canoga Park, CA, USA). CDC is performed on untreated serum and in serum with added dithiothreitol and enhanced with goat IgG anti-human kappa (One Lambda, Canoga Park, CA, USA). All patients are transplanted once a negative CDC T and B cell crossmatch is established, unless they harbor DSA in which FXM is performed. FXM technique used in our laboratory is the one described by ASHI (*ASHI Laboratory Manual*, Fourth Edition, Volume II, 2000). In this scenario, a T cell positive FXM (>40 channel shifts) contraindicates the transplant, while a B cell positive FXM (>100 channel shifts) is approved only for the first transplant.

*Antibody Assessment*. DSA determinations were performed with Luminex LABScreen Single Antigen Beads (LABScreen SAB), class I and class II (One Lambda, Canoga Park, CA, USA), and were systematically obtained as part of the patient's immunological risk profile. Before DSA determination sera were treated with dithiothreitol at a final concentration of 0.005 M, at 37°C during 30 minutes followed by centrifugation at 3000 ×g for 1 minute; after that, the antibody detection described for Luminex using antigen coated beads is followed (*ASHI Laboratory Manual*, Fourth Edition, Volume II, 2000). For this analysis, DSA ≥ 400 MFI was considered positive.

HLA antigen typing and DSA results were obtained in every patient in the Histocompatibility Laboratory database.

*C1q-SAB*. Class I and class II C1q-SAB assays (One Lambda, Canoga Park, CA, USA) were performed in all 41 previously cryopreserved (−70°C) serum samples and according to the manufacturer's directions. Briefly, heat inactivated serum (56°C for 30 minutes) was spiked with 150 mg/mL purified human C1q in HEPES buffer (One Lambda) to ensure equal functional amounts of C1q per sample. Single Antigen Beads were added to the mixture and incubated for 20 minutes at room temperature, followed by addition of phycoerythrin conjugated anti-human C1q. Beads were washed twice and analyzed on a LABScan200 flow analyzer. C1q antibodies MFI threshold for positivity was 400.

Sera sample from the same date the FXM was performed was frozen until tested for C1q assay in October 2013, using the same reagent lot.

### 2.1. Statistical Analysis

Descriptive statistics were used according to the type of analyzed variable. Categorical variables were reported as relative and absolute frequencies. The distribution of continuous variables was evaluated with the Kolmogorov-Smirnov test. Variables with a normal distribution are presented as means and standard deviations, while those with an abnormal distribution are expressed as medians and their interquartile range (IQR). Between-group comparisons of categorical or ordinal variables were performed with Chi^2^ or Fisher's exact test (accordingly). Continuous variables were analyzed with Student's *t*-test or Mann-Whitney's *U* test. Logistic regression analysis was used to predict FXM. A *P* value < 0.05 was considered significant.

We explored the optimum cutoff point to predict a positive FXM by MFI of the immunodominant DSA according to ROC curve. Sensitivity, specificity, and predictive values with 95% CI were calculated for a positive FXM prediction.

The STATA version 11.1 statistical package and Excel 2013 were used.

## 3. Results

The 41 renal transplant candidates included in the study were evaluated between January 2012 and December 2013; five had 2 potential donors, 2 had 3 potential donors, and all were included in the analysis. A total of 50 evaluations of donor/recipient pairs were conducted. The average recipient age was 35 years (±12.72, min–max 8–68), and that of donors was 42.2 years (±11.9, min–max 22–72). Of the 41 studied potential recipients, 23 (56%) were male while 29 (58%) of the potential donors were female.

### 3.1. HLA-Ab and DSA by LABScreen SAB in 41 Renal Transplant Candidates

The 41 analyzed renal transplant candidates had a median number of HLA-Abs of 12 (interquartile range (IQR) 6–28, min–max 2–74) and a median MFI of the dominant HLA-Ab of 10,128 (IQR 3,813–15,407, min–max 696–24,470). The antigenic specificity of these Abs was mostly against HLA-B (*n* = 14, 34%) and HLA-DQ (*n* = 13, 31.7%).

The median number of DSA was 1 (IQR 1-2, min–max 1–6). The DSA-MFI was 2,138 (IQR 1,160–7,147; min–max 493–23,715). The antigen specificity of these HLA-Abs was against class II antigens in 62.0% of cases, homogeneously distributed between HLA-DQ and DR antigens (*n* = 16 and 15, resp.).

### 3.2. Complement Fixing HLA Antibodies (C1q+ Ab)

Over half of the included population had at least one CFAb or C1q+ Ab (*n* = 26, 63.4%). The dominant C1q+ Ab had a C1q-SAB MFI of 14,177 (IQR 8,169–16,359, min–max 614–30161). The MFI of coincidental LABScreen SAB was 13,209 (IQR 10,308–18,562, min–max 832–24470). Most dominant CFAbs (greater MFI in C1q-SAB) were class II (*n* = 18 of 26). Antibodies against HLA-DQ antigens were the most prevalent (*n* = 12 of 26).

**Figure 1:**
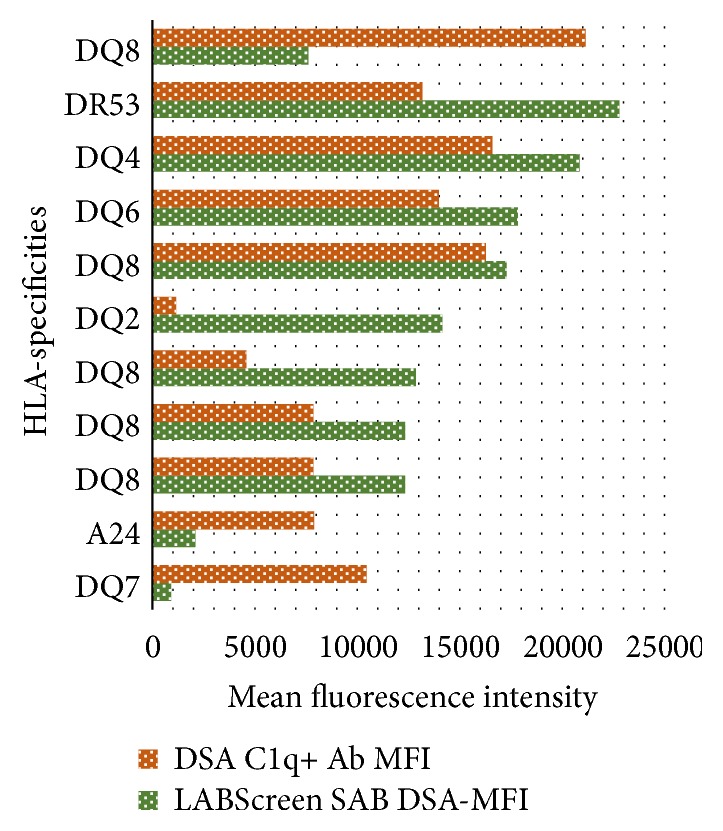
Comparison of the DSA LABScreen SAB MFI and DSA C1q+ Ab in studied patients and HLA antigenic specificities.

Upon evaluation of the donor/recipient pairs (*n* = 50), ten patients (20.0%) had donor-specific C1q+ Ab, and one patient had two antibodies; therefore, 11 DSA C1q+ Abs were detected, with a median C1q MFI of 9,542 (IQR 8,169–14,546, min–max 1,202–17,254). The coincident LABScreen SAB MFI was 14,045 (IQR 12,829–18,562, min–max 956–23,715). Correlation between fluorescence values was poor (*r* ^2^ = 0.19), as shown in Figure 1. Most of these antibodies were against HLA-DQ antigens (*n* = 9), one was against HLA-A antigen, and one was against HLA-DR antigen.

### 3.3. FXM Results

Twenty-five (50%) of a total of 50 FXMs were positive. Sixteen patients were positive against B lymphocytes, 3 were positive against T lymphocytes, and 6 were positive against both cell types. The median FXM channels against B cells were 232 (IQR 157–297, min–max 130–560) and against T cells were 227 channels (IQR 76–259, min–max 44–390). Among the 22 patients with a positive FXM against B lymphocytes, 5 had anti-class I DSA, 10 had anti-class II DSA, and 7 had DSA against both classes. Among the 9 patients with a positive FXM against T lymphocytes, 4 had anti-class I DSA, 1 had anti-class II DSA, and 4 had DSA against both classes.

### 3.4. Factors Associated with a Positive FXM

Table 1 shows the analysis of factors associated with a positive FXM. A greater proportion (nonsignificant) of patients with FXM had a history of previous renal transplantation (50.0% versus 26.1%, *P* = 0.092). It is evident that the presence of DSA C1q+ Ab and the DSA LABScreen SAB MFI, particularly DSA against HLA-A, HLA-DR, and HLA-DQ antigen specificities, are the most significant factors associated with a positive FXM.

**Figure 2:**
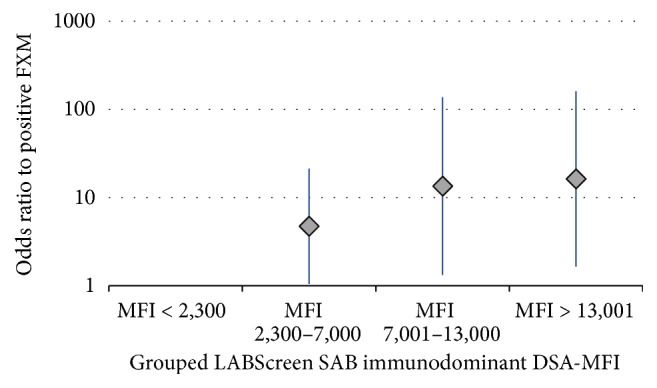
Association of the immunodominant DSA LABScreen SAB MFI with FXM result (logistic regression analysis, expressed odds ratio with 95% confidence interval). DSA w/MFI < 2,300 = reference; MFI 2,300–7,000 = OR 4.75, 1.05–21.35, and *P* = 0.042; MFI 7,001–13,000 = OR 13.58, 1.33–13.45, and *P* = 0.027; and MFI > 13,000 = OR 16.28, 1.65–160.41, and *P* = 0.017.

By logistic regression analysis, the LABScreen SAB MFI of dominant DSA was also significantly associated with a positive FXM (for each 1,000 MFI: OR 1.26, 95% CI 1.06–1.49, and *P* = 0.007). This is also evident when the variable is divided at different cutoff points (Figure 2).

**Figure 3:**
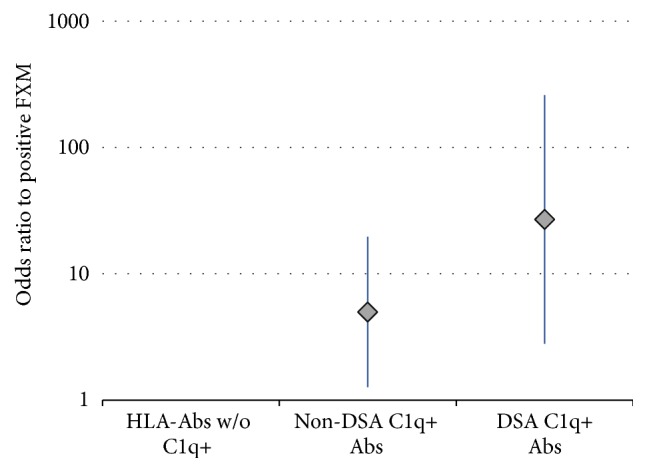
Association of C1q+ Ab (donor-specific and non-donor-specific) with positive FXM (logistic regression analysis, expressed odds ratio with 95% confidence interval). HLA-Ab w/o C1q+ = reference variable; non-DSA C1q+ Ab = OR 5, 1.27–19.68, and *P* = 0.021; and DSA C1q+ Ab = OR 27, 2.80–259.56, and *P* = 0.004.

Also, a positive C1q-SAB assay was associated with a positive FXM (OR 8.14, 2.29–28.9, and *P* = 0.001). When this was analyzed by DSA and non-DSA, both remained significant (Figure 3).

Since the most strongly associated variables to the positive FXM were DSA LABScreen SAB MFI and a positive C1q-SAB assay, we conducted a multivariate logistic regression analysis that revealed an interaction phenomenon. When the patients were grouped according to HLA-Ab LABScreen SAB MFI greater than or under 5000, we detected that the most important association of C1q+ Ab with a positive FXM occurred in the group with DSA LABScreen SAB MFI values <5,000 (OR 5.04, 95% CI 1.1–22.9, and *P* = 0.037) and there was no association in the group with MFI > 5000 (OR 1.0, 95% CI 1.0–0.99, and *P* = 0.485).

After analysis of the median positive channels for T and/or B cells among the 25 positive FXMs in terms of positive or negative C1q-SAB assay, no relation was established. This information is shown in Figure 5, which also specifies the channel shift values of all positive FXMs, those cases with DSA C1q+ Ab among the positive and negative FXM, the MFI for the dominant DSA by LABScreen SAB, and the total number of DSA in each case. Interestingly, even low DSA titers can bind complement (C1q+ Ab) and yield a positive FXM.

### 3.5. FXM Prediction

**Figure 4:**
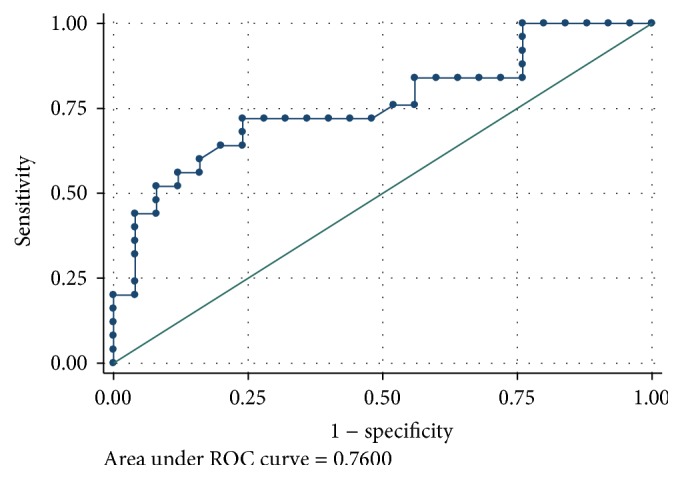
ROC curve of immunodominant DSA LABScreen SAB MFI and FXM. MFI > 2,300 showed the best performance.

**Figure 5:**
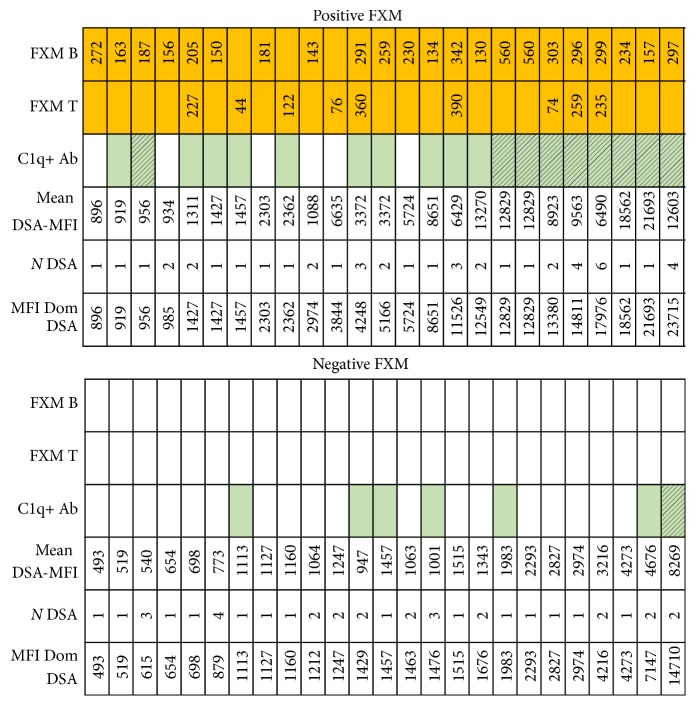
Characteristics of DSA and FXM results. The first two lines show B cell and T cell channel shift results for each positive FXM test; the next line depicts complement fixing HLA-Abs (C1q+ in filled cells) where a dashed square mark indicates DSA C1q+. The next three lines show the mean DSA-SAB MFI, number of DSA, and dominant DSA-SAB MFI. MFI = mean fluorescence intensity, DSA = donor-specific antibodies, *N* = number, C1q+ HLA-Abs = C1q positive HLA antibodies, and FXM = flow crossmatch.

In order to detect the LABScreen SAB MFI cutoff point for DSA that was best associated with FXM, we created ROC curve with the immunodominant DSA. The cutoff point with the greatest sensitivity and specificity was 2,300 (Figure 4).

Table 2 shows the diagnostic performance of the C1q+ Ab (DSA and non-DSA) and LABScreen SAB DSA-MFI > 2,300 and their effect together (C1q+ Ab and MFI > 2300). This last construct yielded the best sensitivity and negative predictive value, while the DSA C1q+ Ab had the best specificity.

## 4. Discussion

The detection of relevant DSA has been limited since the most sensitive and specific method currently used (Luminex-SAB) does not discriminate between clinically relevant antibodies and those that are inert. This is of particular importance in the pretransplant scenario, where antibody generation precedes the donor's antigenic stimulus; in the posttransplant period, the stimulus for the generation of* de novo* antibodies is unequivocally originated by renal graft.

Flow cytometry has significantly contributed to improved patient selection and it is the current standard when selecting transplant candidates. After an appropriate HLA typing and antibody determination by Luminex-SAB, patients with DSA+ and/or FXM+ have been found to be at risk for acute rejection. Some groups have reported that this risk is associated with the presence of DSA even if the FXM is negative [18] while others have found that the presence of DSA detected only by SAB in the context of a negative crossmatch is not associated with an increased risk of acute rejection [19, 20]. However, neither scenario conditions an absolute risk. One of the most representative studies included 21 patients with FXM+ and DSA+ and revealed a greater risk of acute antibody mediated rejection (AMR) when compared to negative FXM patients [21]. Among these patients, 6 (28.5%) developed AMR, while the remaining 15 (66.66%) did not present any antibody mediated events [21]. Due to the broadly documented evidence on the mechanisms of acute rejection in the early posttransplant period and their direct relation with complement activation as a pivotal physiopathological mechanism, the C1q-SAB assay could potentially contribute to the elucidation of the association between complement activation, a positive FXM, and the transplant's outcome.

Our study included the samples of sensitized patients in whom a high prevalence of HLA-Ab capable of binding complement (C1q+ Ab) was documented (*n* = 26, 53.06%); in 10 cases (20%), these HLA-Abs targeted a potential donor's antigenic specificity (DSA C1q+ Ab). In these 10 cases, most were against HLA-DQ (9/11 C1q+ Ab), which is in stark contrast with the original C1q-SAB assay report that detected a greater proportion of anti-class I antibodies capable of binding complement [16]. All 10 cases had a previous renal transplant, which could explain the predominance of HLA-DQ antibodies [22]. Subsequent reports proved that HLA-DQ antigenicity and that of class II antigens in general could generate* de novo* antibodies capable of activating complement, leading to AMR and graft loss [23]. It is important to mention that DP specificity was no typing, which constitutes a limitation of the present analysis, although the relevance of HLA-Ab versus DP specificities has not been clearly recognized as deleterious at this moment [24]. In clinical practice in our particular setting, patients with DSA against a potential living donor and a negative AHG-CDC-XM are not infrequent. Following current guidelines, these patients should be evaluated by FXM to determine whether the transplant may proceed without the need for any desensitization maneuvers [6].

We observed that 25 (50%) of our FXMs were positive. Of these, only 9 (36%) had DSA C1q+ Ab. This number reflected the high specificity in predicting FXM+ (95.8%). Ten more patients with a positive FXM were non-DSA C1q+ Ab. This same situation occurred in 6 of 25 patients with a negative FXM. Compared to patients with no CFAbs, both the non-DSA C1q+ Ab group and the DSA C1q+ Ab group were at significant risk for a positive FXM (OR 5, 95% CI 1.27–19.68, and *P* = 0.021, and OR 27, 95% CI 2.80–259.56, and *P* = 0.004, resp.). Cases with DSA C1q+ Ab and negative DSA by IgG-SAB have been attributed to IgM antibodies or a prozone phenomenon; in this series, however, we did not detect any cases with these characteristics. All patients included in this study had DSA that can explain the positive FXM. To attribute this positivity to a non-DSA C1q+ Ab cannot be clearly biologically sustained. Complement activating of antibodies against donor-specific epitopes may be a possibility. Several studies have emphasized the importance of antibody specificity against epitopes that may be shared by more than one HLA antigen and this would certainly denote a limitation to serologic determination by Luminex. Moreover, it is also possible that we are detecting a more immunologically robust group of patients with the ability to generate more functional antibodies. In either case, this study is not designed to affirm or reject these proposals.

Based on these findings and being well aware of the study's methodology limitations, particularly in terms of its pretransplant and cross-sectional design, we must emphasize the clinical usefulness of our results. Above all, only in 9 of 25 patients with a positive FXM could an explanation hinge on complement activation. Flow cytometry is known to detect donor-specific antibodies that bind to the cell membrane regardless of their functionality. However, the clinical significance of FXM+ in the absence of complement fixing DSA remains to be determined. We must emphasize that two-thirds of FXM+ and DSA+ patients will never develop early AMR phenomena [21]. However, complement-independent mechanisms of endothelial cell damage by anti-HLA antibodies have been proposed. HLA class I antibody binding to endothelial cell activated the mTOR pathway via Src/FAK/paxillin complex, leading to Akt, ERK, and S6RP phosphorylation in a manner dependent on the association of the HLA class I protein with integrin [25, 26]. Recently, an experimental model of HLA-DR antibody stimulation of microvascular endothelial cells (ECs) and allograft damage showed activation of ECs with F(Ab′)2 fragment of HLA-DR Ab which led to phosphorylation of Akt, ERK, and MEK and increased in IL-6 production by ECs cocultured with allogenic PBMCs in an Akt-dependent manner. Furthermore, preactivation of ECs with anti-HLA-DR antibody redirected endothelial cell allogenicity toward a proinflammatory response by decreasing amplification of functional Treg and by further increasing IL-6-dependent Th17 expansion [27]. Notably, both the presence of DSA C1q+ Ab and the LABScreen SAB fluorescence values were significantly related to FXM+. Moreover, 9 of the 10 patients with DSA C1q+ Ab had dominant DSA fluorescence values above 10,000 and only one case had MFI of 956. On the other hand, two patients had dominant DSA > 10,000 but were negative for C1q-SAB.

Definitely, the C1q+ SAB value as a pretransplant screening tool will have to be further evaluated, initially, in pretransplant serum samples of transplanted patients with DSA, determining their FXM status and their need for desensitization strategies if it was required. In case an adequate association with the short-term transplant outcome is detected, a randomized clinical trial based on C1q-SAB results should be posited. In this regard, circulating DSA detected on bead arrays was recently evaluated and whether detection of complement fixation using C1q, C4d, or C3d assays in parallel to IgG MFI allowed for improved prediction of AMR was investigated and it was found that IgG MFI add predictive accuracy for silent AMR in DSA+ long-term recipients, and detecting complement fixation did not add independent diagnostic advantage [28].

To our knowledge, this is the first study attempting to associate FXM with the C1q-SAB assay, and it could well contribute to the elucidation of potential interacting mechanisms.

In conclusion C1q+ Ab and LABScreen SAB DSA-MFI were significantly associated with FXM. DSA C1q+ Ab was highly specific but with low sensitivity. The clinical significance of FXM+ in the absence of complement fixing DSA remains to be determined.

## Figures and Tables

**Table 1:** Factors associated with a positive FXM. DSA determinations are expressed according to the LABScreen SAB results. MM = mismatches.

	All samples (*n* = 50)	Positive FXM (*n* = 25)	Negative FXM (*n* = 25)	*P* value
Recipient age, m (SD)	35.0 ± 12.7	33.8 ± 11.2	36.2 ± 14.1	0.510
Recipient male gender, *n* (%)	23 (46.0)	9 (36.0)	14 (56.0)	0.156
Donor age, m (SD)	42.2 ± 11.9	43.7 ± 10.6	40.7 ± 13.1	0.385
Donor male gender, *n* (%)	21 (42.0)	12 (48.0)	9 (36.0)	0.390
Previous transplant, *n* (%)	18 (38.3)	12 (50.0)	6 (26.1)	0.092
HLA MM, med (IQR)	4 (4–6)	4 (3–6)	4 (4–6)	0.889
HLA MM DR/DQ, med (IQR)	2 (1-2)	2 (2-2)	2 (1-2)	0.495
% PRA class I, med (min–max)	15 (0–90)	14.0 (2–90)	16 (0–60)	0.259
% PRA class II, med (min–max)	10 (4–24)	13 (1–89)	9 (0–100)	0.466
Class I DSA, *n* (%)	29 (58.0)	14 (56.0)	15 (60.0)	0.774
Class II DSA, *n* (%)	35 (70.0)	18 (72.0)	17 (68.0)	0.758

*HLA antibodies analyzed by LABScreen SAB*				
*n* of DSA, m (min–max)	1.72 (1–6)	1.84 (1–6)	1.6 (1–4)	0.769
MFI DSA, med (IQR)	2,138 (1,160–7,147)	5,166 (1,457–12,829)	1,457 (1,113–2,293)	0.001
*n* of all HLA-Ab, med (IQR)	12 (6–28)	12 (10–26)	8 (4–28)	0.164
MFI all HLA-Ab, med (IQR)	10,128 (3,813–15,407)	13,312 (5,681–18,666)	3,937 (2,192–13,312)	0.006
HLA-A DSA, *n* (%)	16 (32.0)	7 (28.0)	9 (36.0)	0.544
MFI DSA HLA-A, med (IQR)	1,294.5 (763–4,155.5)	4,467 (3,141–11,526)	828 (664–1,113)	0.003
HLA-B DSA, *n* (%)	9 (18.0)	5 (20.0)	4 (16.0)	1.00
MFI DSA HLA-B, med (IQR)	1,427 (759–2,669)	2,669 (1,427–5,670)	838 (706.5–1,190)	0.141
HLA-C DSA, *n* (%)	12 (24.0)	5 (20.0)	7 (28.0)	0.508
MFI DSA HLA-C, med (IQR)	1,866 (843.5–2,739.5)	2,362 (2,303–2,505)	1,247 (612–2,974)	0.329
HLA-DR DSA, *n* (%)	20 (40.0)	9 (36.0)	11 (44.0)	0.564
MFI DSA HLA-DR, med (IQR)	1,752.5 (1,177.5–3,986)	5,145 (1,457–8,651)	1,515 (1,127–2,216)	0.048
HLA-DQ DSA, *n* (%)	21 (42.0)	13 (52.0)	7 (32.0)	0.152
MFI DSA HLA-DQ, med (IQR)	7,147 (1,247–14,710)	12,829 (1,578–17,976)	1,615 (908.5–5,710)	0.042

*HLA antibodies by C1q assay*				
All C1q+ Ab, *n* (%)	26 (53.06)	19 (76.0)	7 (28.0)	0.001
DSA C1q+ Ab, *n* (%)	10 (20.41)	9 (36.0)	1 (4.0)	0.005
Non-DSA C1q+ Ab, *n* (%)	16 (32.65)	10 (40)	6 (24.0)	0.023
HLA-A C1q+ Ab, *n* (%)	6 (12.0)	4 (16.0)	2 (8.0)	0.667
HLA-B C1q+ Ab, *n* (%)	8 (16.0)	6 (24.0)	2 (8.0)	0.247
HLA-C C1q+ Ab, *n* (%)	3 (6.0)	1 (4.0)	2 (8.0)	1.0
HLA-DP C1q+ Ab, *n* (%)	6 (6.0)	3 (12.0)	0	0.235
HLA-DQ C1q+ Ab, *n* (%)	19 (38.0)	16 (64.0)	3 (12.0)	<0.001
HLA-DR C1q+ Ab, *n* (%)	8 (16.0)	7 (28.0)	1 (4.0)	0.049

**Table 2:** Sensitivity, specificity, PPV, and NPV of DSA C1q+ Ab; C1q+ Ab (DSA and non-DSA); DSA LABScreen SAB MFI > 2,300; and DSA > 2,300 plus C1q+ Ab (DSA and non-DSA).

	DSA C1q+ Ab	C1q+ Ab (DSA & non-DSA)	DSA-MFI > 2300	DSA > 2300 plus C1q+ Ab (DSA & non-DSA)
Sensitivity	36% (15.1–56.8)	76.0 (57.2–94.7)	72.0 (52.4–91.6)	92.0 (79.3–100)
Specificity	95.8% (85.7–100)	70.8 (50.5–91.1)	75.0 (55.5–94.4)	54.1 (32.1–76.1)
PPV	90 (66.4–100)	73.08 (54.1–92.05)	73.47 (55.5–94.4)	67.65 (50.45–84.84)
NPV	58.9 (42.2–75.6)	73.91 (53.7–94.03)	72.0 (52.4–91.6)	86.67 (66.13–100)

## References

[1] Patel R., Terasaki P. I. (1969). Significance of the positive crossmatch test in kidney transplantation. *The New England Journal of Medicine*.

[2] Pei R., Lee J.-H., Chen T., Rojo S., Terasaki P. I. (1999). Flow cytometric detection of HLA antibodies using a spectrum of microbeads. *Human Immunology*.

[3] Gibney E. M., Cagle L. R., Freed B., Warnell S. E., Chan L., Wiseman A. C. (2006). Detection of donor-specific antibodies using HLA-coated microspheres: another tool for kidney transplant risk stratification. *Nephrology Dialysis Transplantation*.

[4] Bray R. A., Gebel H. M. (2009). Strategies for human leukocyte antigen antibody detection. *Current Opinion in Organ Transplantation*.

[5] Zachary A. A., Sholander J. T., Houp J. A., Leffell M. S. (2009). Using real data for a virtual crossmatch. *Human Immunology*.

[6] Tait B. D., Süsal C., Gebel H. M. (2013). Consensus guidelines on the testing and clinical management issues associated with HLA and non-HLA antibodies in transplantation. *Transplantation*.

[7] Feucht H. E., Schneeberger H., Hillebrand G. (1993). Capillary deposition of C4d complement fragment and early renal graft loss. *Kidney International*.

[8] Crespo M., Pascual M., Tolkoff-Rubin N. (2001). Acute humoral rejection in renal allograft recipients: I. Incidence, serology and clinical characteristics. *Transplantation*.

[9] Wahrmann M., Exner M., Regele H. (2003). Flow cytometry based detection of HLA alloantibody mediated classical complement activation. *Journal of Immunological Methods*.

[10] Wahrmann M., Exner M., Haidbauer B. (2005). [C4d]FlowPRA screening—a specific assay for selective detection of complement-activating anti-HLA alloantibodies. *Human Immunology*.

[11] Bartel G., Wahrmann M., Exner M. (2008). In vitro detection of C4d-fixing HLA alloantibodies: associations with capillary C4d deposition in kidney allografts. *American Journal of Transplantation*.

[12] Wahrmann M., Bartel G., Exner M. (2009). Clinical relevance of preformed C4d-fixing and non-C4d-fixing HLA single antigen reactivity in renal allograft recipients. *Transplant International*.

[13] Smith J. D., Hamour I. M., Banner N. R., Rose M. L. (2007). C4d fixing, luminex binding antibodies—a new tool for prediction of graft failure after heart transplantation. *American Journal of Transplantation*.

[14] Honger G., Wahrmann M., Amico P., Hopfer H., Böhmig G. A., Schaub S. (2010). C4d-fixing capability of low-level donor-specific HLA antibodies is not predictive for early antibody-mediated rejection. *Transplantation*.

[15] Wahrmann M., Exner M., Schillinger M. (2006). Pivotal role of complement-fixing HLA alloantibodies in presensitized kidney allograft recipients. *American Journal of Transplantation*.

[16] Chen G., Sequeira F., Tyan D. B. (2011). Novel C1q assay reveals a clinically relevant subset of human leukocyte antigen antibodies independent of immunoglobulin G strength on single antigen beads. *Human Immunology*.

[17] Chen G., Sequeira F., Tyan D. (2013). A new combo-flow crossmatch (CFMX) for simultaneous detection of IgG and complement fixing antibodies (CFABS). *Abstracts/Human Immunology*.

[18] Patel A. M., Pancoska C., Mulgaonkar S., Weng F. L. (2007). Renal transplantation in patients with pre-transplant donor-specific antibodies and negative flow cytometry crossmatches. *American Journal of Transplantation*.

[19] Gupta A., Iveson V., Varagunam M., Bodger S., Sinnott P., Thuraisingham R. C. (2008). Pretransplant donor-specific antibodies in cytotoxic negative crossmatch kidney transplants: are they relevant?. *Transplantation*.

[20] Reinsmoen N. L., Lai C.-H., Vo A. (2008). Acceptable donor-specific antibody levels allowing for successful deceased and living donor kidney transplantation after desensitization therapy. *Transplantation*.

[21] Couzi L., Araujo C., Guidicelli G. (2011). Interpretation of positive flow cytometric crossmatch in the era of the single-antigen bead assay. *Transplantation*.

[22] Duquesnoy R. J., Awadalla Y., Lomago J. (2008). Retransplant candidates have donor-specific antibodies that react with structurally defined HLA-DR,DQ,DP epitopes. *Transplant Immunology*.

[23] Freitas M. C. S., Rebellato L. M., Ozawa M. (2013). The role of immunoglobulin-G subclasses and C1q in de novo HLA-DQ donor-specific antibody kidney transplantation outcomes. *Transplantation*.

[24] Redondo-Pachón D., Pascual J., Pérez-Sáez M. J. (2016). Impact of preformed and de novo anti-HLA DP antibodies in renal allograft survival. *Transplant Immunology*.

[25] Lepin E. J., Reed E. F. (2004). Complement-independent mechanisms of antigraft antibodies in transplant arteriosclerosis and accommodation. *Current Opinion in Organ Transplantation*.

[26] Jindra P. T., Jin Y.-P., Rozengurt E., Reed E. F. (2008). HLA class I antibody-mediated endothelial cell proliferation via the mTOR pathway. *Journal of Immunology*.

[27] Lion J., Taflin C., Cross A. R. (2016). HLA class II antibody activation of endothelial cells promotes Th17 and disrupts regulatory T lymphocyte expansion. *American Journal of Transplantation*.

[28] Eskandary F., Bond G., Kozakowski N. (2016). Diagnostic contribution of donor-specific antibody characteristics to uncover late silent antibody-mediated rejection-results of a cross-sectional screening study. *Transplantation*.

